# Sagnac with Double-Sense Twisted Low-Birefringence Standard Fiber as Vibration Sensor

**DOI:** 10.3390/s22218557

**Published:** 2022-11-07

**Authors:** Héctor Santiago-Hernández, Anuar Benjamín Beltrán-González, Azael Mora-Nuñez, Beethoven Bravo-Medina, Olivier Pottiez

**Affiliations:** 1 Departamento de Ingeniería Electro-Fotónica, Universidad de Guadalajara (UDG), Blvd. Gral. Marcelino García Barragán 1421, Guadalajara 44430, Jalisco, Mexico; 2 Centro de Investigaciones en Óptica (CIO), Loma del Bosque 115, Col. Lomas del Campestre, León 37150, Guanajuato, Mexico

**Keywords:** Sagnac interferometer, vibration sensor, optical fiber

## Abstract

In this work, we study a double-sense twisted low-birefringence Sagnac loop structure as a sound/vibration sensing device. We study the relation between the adjustments of a wave retarder inside the loop (which allows controlling the transmission characteristic to deliver 10, 100, and 300 μW average power at the output of the system) and the response of the Sagnac sensor to vibration frequencies ranging from 0 to 22 kHz. For a 300 m loop Sagnac, two sets of experiments were carried out, playing at the same time all the sound frequencies mixed for ∼1 s, and playing a sweep of frequencies for 30 s. In both cases, the time- and frequency-domain transmission amplitudes are larger for an average power of 10 μW, and smaller for an average power of 300 μW. For mixed frequencies, the Fourier analysis shows that the Sagnac response is larger for low frequencies (from 0 to ∼5 kHz) than for high frequencies (from ∼5 kHz to ∼22 kHz). For a sweep of frequencies, the results reveal that the interferometer perceives all frequencies. However, beyond ∼2.5 kHz, harmonics are present each ∼50 Hz, revealing that some resonances are present. The results about the influence of the power transmission through the polarizer and power emission of laser diode (LD) on the Sagnac interferometer response at high frequencies reveal that our system is robust, and the results are highly reproducible, and harmonics do not depend on the state of polarization at the input of the Sagnac interferometer. Furthermore, increasing the LD output power from 5 mW to 67.5 mW allows us to eliminate noisy signals at the system output. in our setup, the minimum sound level detected was 56 dB. On the other hand, the experimental results of a 10 m loop OFSI reveal that the response at low frequencies (1.5 kHz to 5 kHz) is minor compared with the 300 m loop OFSI. However, the response at high frequencies is low but still enables the detection of these frequencies, yielding the possibility of tuning the response of the vibration sensor by varying the length of the Sagnac loop.

## 1. Introduction

Optical fibers constitute an extraordinary sensing platform because of their specific advantages, such as small size, relatively low cost, and immunity to electromagnetic interference. Additionally, fiber optic interferometers have been widely investigated for sensing various physical parameters, including temperature, strain, pressure, and refractive index, because they provide high sensitivity and accurate results [1,2]. In particular, the fiber Sagnac interferometer is a simple and robust structure fabricated by forming a fiber loop between the output ports of a directional coupler. In this scheme, the two optical signals are guided in opposite directions in the loop. The reflectivity of the device depends on the coupling ratio and loop birefringence, which are wavelength-dependent [3,4]. Fiber loop reflectors have been widely deployed in multiple and practical scenarios as sensors [2,5], multiplexers [4,6], or for the generation and characterization of laser dynamics [7,8,9,10].

Recently, there has been increasing interest in the study of fiber optic sensors for acoustic/vibration sensing, motivated by their significant importance in scientific measurements and engineering applications [11,12,13]. The Optical Fiber Sagnac Interferometer (OFSI) has the advantages of small size, lightweight, easy laying, high sensitivity, anti-electromagnetic interference, intrinsic safety, etc. However, the OFSIs reported for vibration sensing are composed of special fibers or elaborate setups: In [14], a high birefringence (Hi-Bi) Polarization Maintaining (PM) fiber is used as the sensing element for static axis strain and dynamic vibration measurements. In [15], a fiber vibration sensor system was constructed with a light-emitting diode and a balanced PM fiber Sagnac interferometer, in which one of the PM fibers was used as a sensing cable and the other as a reference cable. In [16], a real-time distributed optical fiber vibration sensing prototype based on the Sagnac interference in conjunction with Optical Time Domain Reflectometry (OTDR) was developed. In [17], a vibration location method with nested pulses is proposed for a distributed optical fiber vibration sensing system. Additionally, several studies have shown a wide range of situations in which the OFSI systems allow sensing vibrations. In [18], a vibration sensor based on a Sagnac interferometer and Fiber Ring Laser (FRL) is proposed for fault diagnosis of bearing, and a sensing system of vibration is demonstrated experimentally. In [19], a Distributed Optical Fiber Fensor System (DOFS) uses acoustic wave emission phenomena to detect and locate electric discharges in electric machines and insulated electrical cables. In [20], an Optical Fiber Sagnac Interferometer system is proposed for electrical cable safety evaluation by partial discharge ultrasound detection.

In this work, we study a simple and low-cost Sagnac with double-sense twisted low-birefringence standard fiber as a stable, robust, and highly reproducible acoustic/vibration sensor for the first time, to the best of our knowledge. The double-sense twisted fiber scheme displays several benefits by reducing the linear and circular birefringence effects, resulting in a wavelength-independent system, a transmission of low-power radiation, maintaining the state of polarization, and no temperature effects [10,21,22]. In our setup, we implement strict control of the polarization of light. We can enhance the sensibility of OFSI to sense acoustic/vibration phenomena. We, especially, seek to generate knowledge related to the interaction of vibrations with the optical response of a Saganac interferometer.

## 2. Experimental Setup and Principles

**Figure 1:**
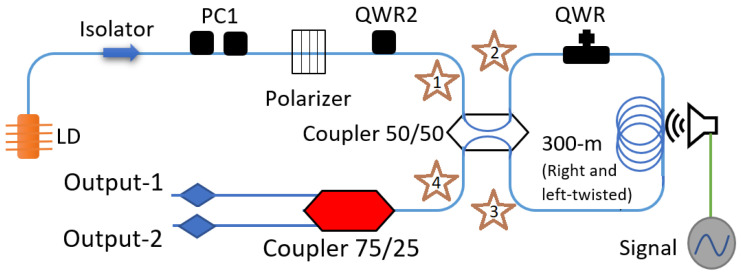
Schematic diagram of the OFSI.

The experimental setup, shown in Figure 1, is a Sagnac interferometer system that allows strict control of polarization. The system is composed of a Laser Diode (LD) emitting at 1550 nm wavelength (Thorlabs, Model: FPL1009), an isolator to prevent the optical power reflected by the Sagnac from damaging the LD. A Polarization Controller (PC1), which consists of two retarder plates of λ/2 (HWR1) and λ/4 (QWR1), respectively, is then inserted: It is used to maximize the power transmission through the polarizer. The polarizer and QWR2 were used to ensure circular polarization at the OFSI input. The Sagnac interferometer is a polarization-imbalanced scheme. It is formed by a 50/50 coupler, an in-line Polarization Controller adjusted as a quarter wave retarder (QWR) which, through pressure applied on the fiber, converts linear polarization into any desired ellipticity [23]. The loop also includes two 150 m pieces of highly right- and left-twisted (7 turns/m) SMF-28 optical fiber to build a wavelength-independent system [10]. The 300 m of fiber was coiled on a drum with a 65 cm diameter. Finally, a 75/25 coupler is used to provide two output ports.

A Data Acquisition (DAQ), model NI MyDAQ (Part number: 195509D-01L), was used as a function generator and oscilloscope. The DAQ allows us to generate several frequencies for our analysis. The signal consists of several frequencies (0–22,000 Hz) playing for ∼1 s through a speaker near the fiber Sagnac loop. The loudspeaker volume level was set at 96 dB, measured by a commercial instrument: analog sound meter, extech, model 407706. The frequencies were spaced every 100 Hz. The intensity response of the scheme to vibration was detected by a 5 GHz photodetector with 100 kHz NI MyDAQ; an analog input converts a voltage level into a digital value that can be stored and processed on a computer. At the output of the setup (port 4, marked with a star in Figure 1) was connected a 75/25 coupler, where we could characterize the vibration response of the OFSI by measuring simultaneously the average power at output 1 (75%), and time-domain modulations at output 2 (25%).

**Figure 2:**
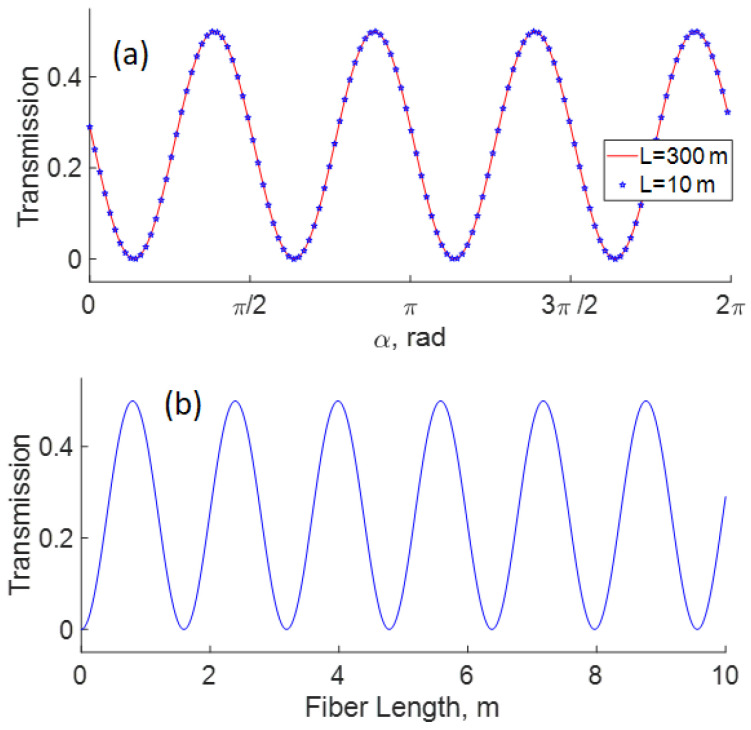
Dependence of Sagnac transmission on (**a**) angle α of QWR in the Sagnac loop, (**b**) loop length (for α = 0). The traces are calculated by using Equation (1), with q=14π, h=0.13, and n=1.45.

The OFSI scheme described above originates from the seminal work by Kuzin et al., which focused on polarization-imbalanced nonlinear Sagnac devices [24]. In the particular case of the symmetrical coupler (r = 0.5) and a QWR, the power transmission, defined as the ratio between the output power and the input power, is given by [25]:

(1) $$T=\frac{1}{4}\left[1-\cos\left(2\beta -4\alpha \right)\right],\beta =\frac{hqL}{2n},$$

where α is the angle (measured from the horizontal plane) of QWR inserted in the loop, *q* is the fiber twist rate (in radians per unit length), the circular birefringence for silica fiber h≈0.13 [26], *L* is the fiber length, and *n* its refractive index (n≈1.45 at 1550 nm). Some implications of Equation (1) are depicted in Figure 2. It is important to note that the control of transmission by using a QWR is possible independently of the length of the Sagnac loop, as shown in Figure 2a. The response of the interferometer also depends on the length of the Sagnac fiber loop, as shown in Figure 2b. In the same figure, it is possible to appreciate that the period of transmission is ΔL≈1.6 m.

**Figure 3:**
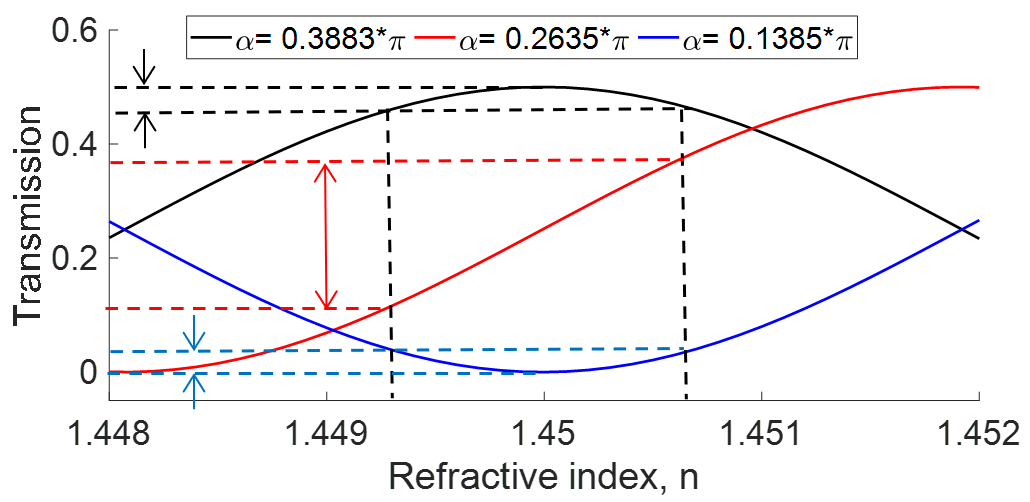
Dependence of Sagnac transmission on refractive index in the loop, for different values of QWR angle α. Double arrows illustrate the amplitude of transmission variations resulting from a given index fluctuation. The symbol (*) denotes multiplication.

For a 300 m Sagnac loop, and α value fixed, we can modify the OFSI transmission if the refractive index in Equation (1) is varied, as shown in Figure 3. Let us first assume that the transmission is adjusted at its minimum (α=0.1385π) or maximum value (α=0.3883π) for n=1.45 (*T* = 0 and 0.5 for the blue and black traces, respectively). If now small variations of refractive index take place, significant changes in transmission appear, as shown in Figure 3. If the transmission is adjusted to a minimal value by angle α, then any variation in the refractive index implies that the transmission increases. On the contrary, if the transmission is adjusted by QWR (angle α) for maximum transmission, then any variation of the refractive index implies that transmission decreases. However, the largest transmission changes for a given refractive index variation are obtained for adjustments intermediate between maximal and minimal transmission (see the red curve in Figure 3).

The cylindrical structure of an optical fiber supports discrete sets of longitudinal, radial, torsional, and flexural elastic vibrations [27]. Furthermore, the vibration modes in the optical fiber have properties suitable for interaction with a sound field surrounding the fiber, producing stress-birefringence patterns in the same fiber [28]. In this context, it is possible to modify the refractive index of an optical fiber by the sound surrounding the optical fiber. In this work, we propose a very simple OFSI with strict control of birefringence as a vibration sensor that allows tuning the amplitude of sensed vibrations.

## 3. Results and Discussions

With the LD at 50 mA current (5 mW optical power) and the in-line Polarization Controller tuned as a quarter-wave retarder by the screw, we ensure a high transmission through the polarizer by adjusting the angle of plates in PC1. Subsequently, QWR2 was adjusted to obtain circular polarization at the input of the OFSI. First, to analyze the response of the interferometer to different vibrational frequencies for different OFSI adjustments, we modify the transmission from a minimum to a maximum value by changing the angle α of QWR, see Figure 2a. The minimum and maximum average power detected were 0.08 μW and 300 μW at 65 and 110 degrees relative to the horizontal plane, respectively. However, experimental measurements showed variations of ∼10 μW each 1 degree in the range of ∼85 to ∼95 degrees. Specifically, the measurements were recorded for three different QWR adjustments, corresponding to 75, 88, and 110 degrees for 10, 100, and 300 μW of average power, respectively, measured at the 75% port of the output coupler connected at the output of the interferometer. The latter value of the average power was obtained when the OFSI was adjusted for maximal transmission (black curve in Figure 3), while the other values correspond to adjustments intermediate between minimal and maximal transmission.

**Figure 4:**
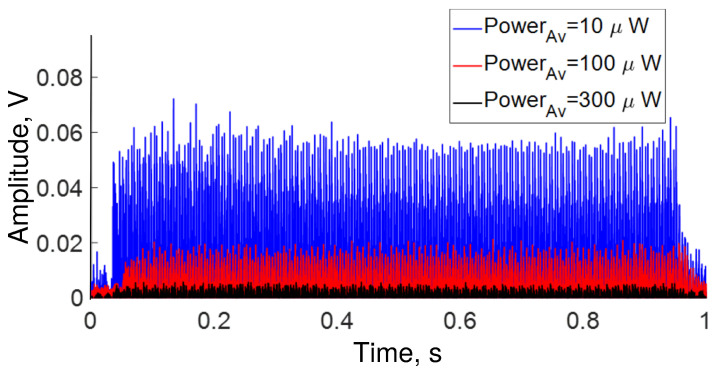
All frequencies detected during ∼1 s by OFSI for several average power values adjusted by QWR angle α (see Figure 1).

In the first experiment, we simultaneously played all frequencies from 0 to 22 kHz during ∼1 s, then the OFSI response was analyzed. The temporal response is shown in Figure 4. The blue, red, and black traces represent the transmission at 10, 100, and 300 μW of average power, respectively. In the same figure, it can be seen that the amplitude of the transmission response is higher for an average power of 10 μW, whereas the amplitude of transmission is smaller for an average power of 300 μW. As expected, the smallest response is obtained in the case of 300 μW (QWR adjusted for maximal transmission), whereas the largest responses were observed for adjustments intermediate between maximal and minimal transmissions. Although, in theory, the largest response would be expected to be at 100 μW instead of 10 μW, some deviation with respect to the ideal sinusoidal transmission characteristic can account for the observed results. Note also that although 10 μW (at 75° QWR angle) is a small value of the average power compared to the maximum, its position is significantly shifted from the minimum (0.08 μW at 65°), where a small response would be expected.

**Figure 5:**
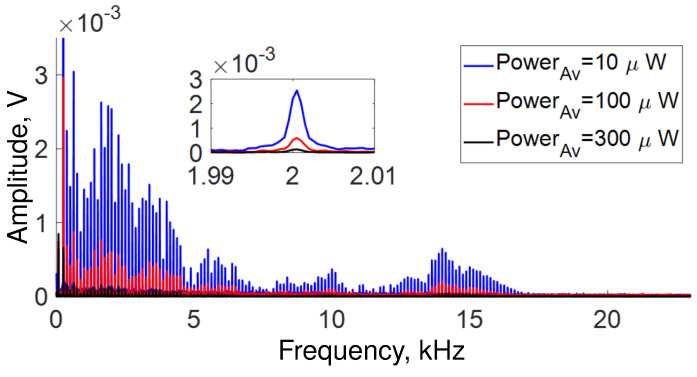
All frequencies detected in our setup for several average power adjusted by the angle α of QWR (see Figure 1). Inset a zoom of frequencies at 2 kHz.

The frequencies detected in our setup are depicted in Figure 5. However, the intensity of the frequency components changes in the same way as the amplitude shown in Figure 4. Namely, the intensity decreases as the average power increases, as shown in the inset of the same Figure 5. Furthermore, it is possible to appreciate in the same figure that the response of the OFSI varies significantly for different vibrational frequencies. Our setup response is better for low frequencies (from 0 Hz to ∼5 kHz) than for high frequencies (from ∼5 kHz to ∼22 kHz). The intensity of the response is not a simple decay with frequency, and the frequencies that were best detected in our setup are centered at 0.6255 kHz, 1.8755 kHz, 5.5 kHz, 10 kHz, and 14 kHz. It is important to note that these frequencies where the acoustic response is maximal seem to coincide with a fundamental frequency *f* = 0.6255 kHz and its 3rd, 9th and 16th harmonics, suggesting that a resonance phenomenon is present.

**Figure 6:**
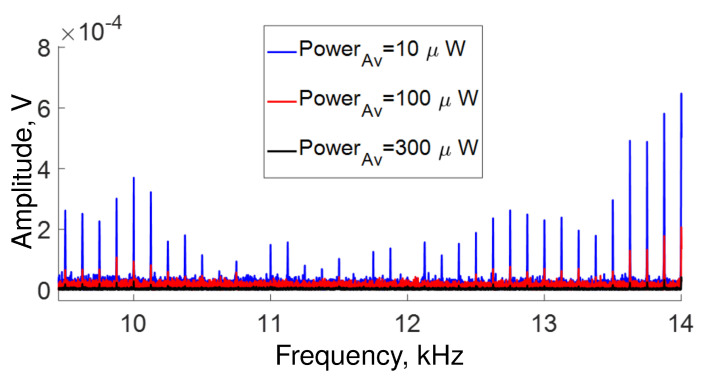
Low-response region for frequencies detected during 1 s in our setup for several average power measured at the output and adjusted by the angle α of QWR (see Figure 1).

The low-response region (from ∼9.5 kHz to ∼14 kHz) is depicted in Figure 6. Although the interferometer has low transmission in this region, it is possible to detect vibration frequencies. However, the same figure shows that some frequencies vanish at ∼10.8 kHz, ∼11.6 kHz and ∼12 kHz for low and high average transmitted power values, indicating that destructive interference may occur at these frequencies.

**Figure 7:**
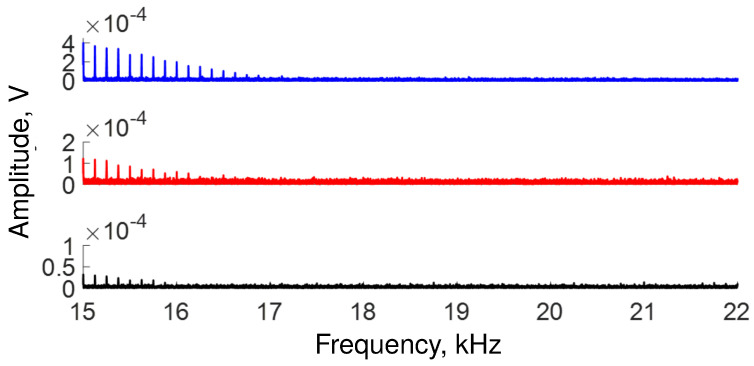
The OFSI response decays gradually at high frequencies. Specifically, the signal disappears at ∼17, ∼16.5, and ∼16 kHz, for blue, red, and black traces, respectively.

The lowest response of our OFSI occurs at the high-frequency end of the spectrum in the region that ranges from ∼15 kHz to ∼22 kHz, as shown in Figure 7. The blue, red, and black traces show the response at 10, 100, and 300 μW of average power, respectively. The attenuation of the signal described by this figure implies that some signals are not detected. Specifically, the traces show that, at high frequencies, the signal disappears as the transmission response is no longer detected.

**Figure 8:**
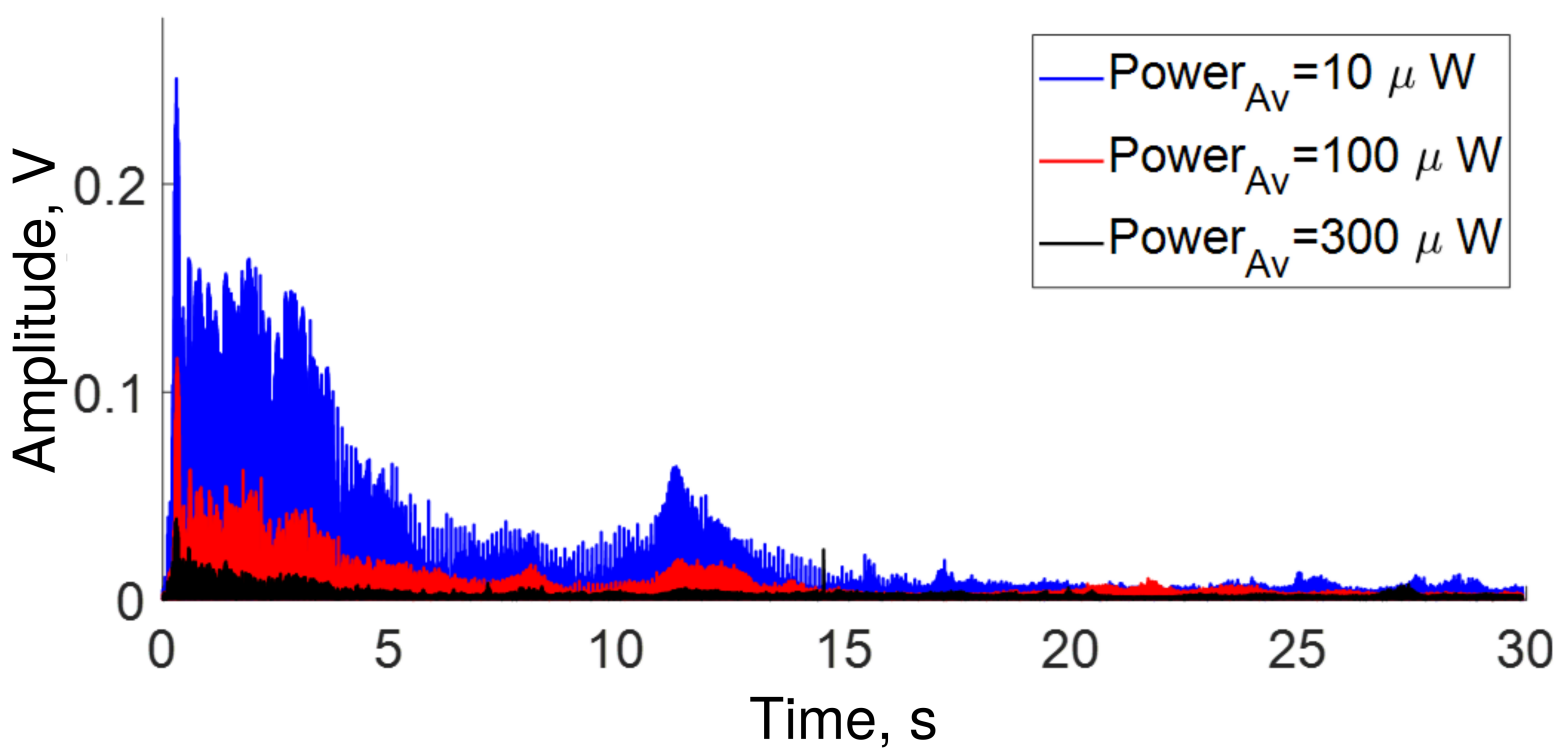
Response of OFSI for a sweep of frequencies from 0 kHz to 23 kHz over 30 s for several average power values adjusted by the angle α of the QWR (see Figure 1).

**Figure 9:**
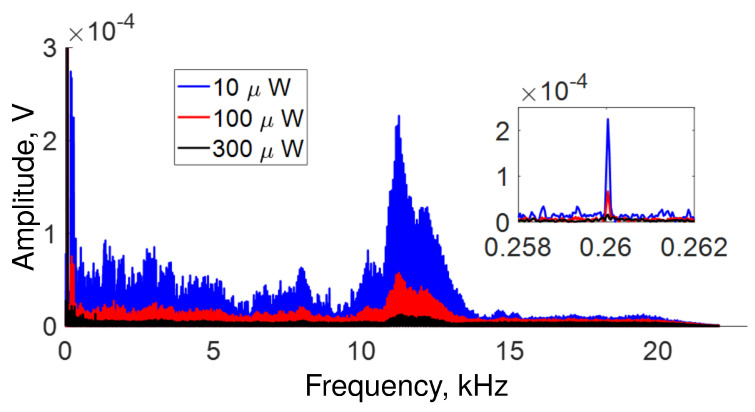
All frequencies detected in our setup for several values of average power adjusted by the angle α of QWR (see Figure 1). The inset shows a zoom on frequencies around 0.26 kHz.

**Figure 10:**
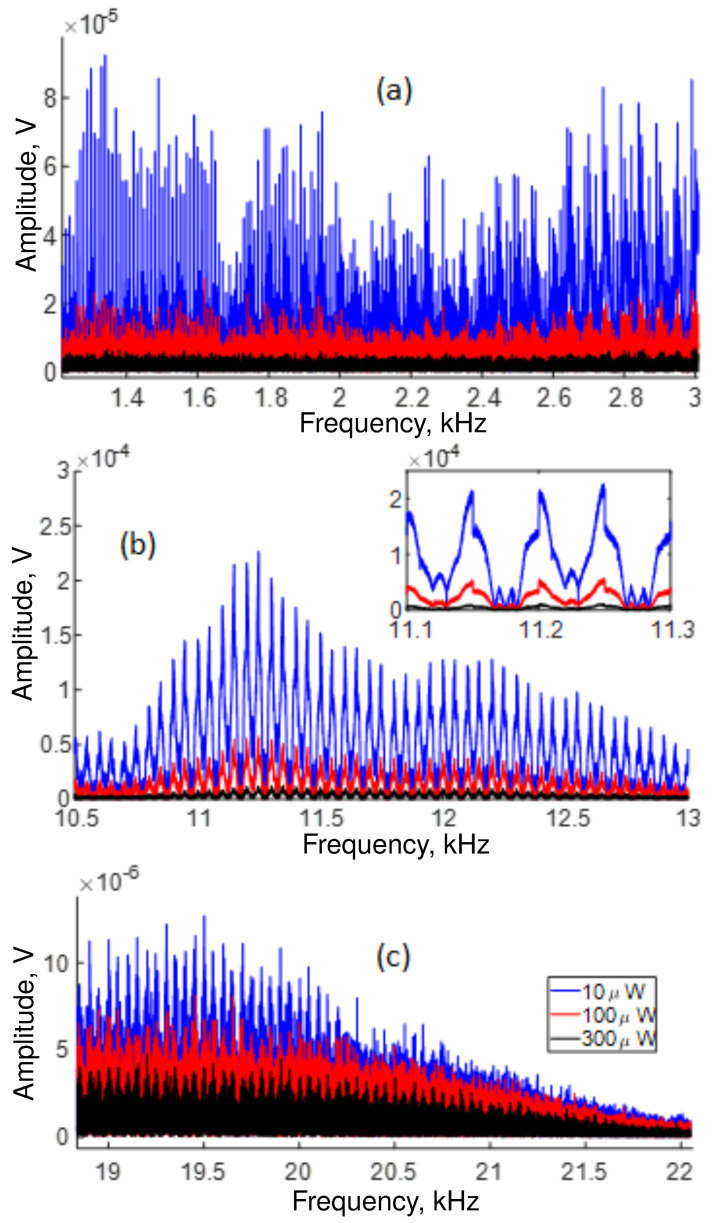
Frequency-dependent response of the system for several average power adjusted by the angle α of QWR (see Figure 1): (**a**) in the 2 kHz region, (**b**) around 11.2 kHz; (**c**) at high frequencies.

In a second experiment, we play a sweep of frequencies from 0 to 22 kHz over 30 s. The temporal and frequential responses of the OFSI are shown in Figure 8, Figure 9 and Figure 10. The traces in Figure 8 describe a behavior similar to that of Figure 4. Namely, for low average power (10 μW), the transmission response is high, in contrast, for high average power (300 μW), the response is low. In the same figure, it can be seen that the response at each frequency is different. In other words, the OFSI has a higher sensitivity for low frequencies than for high frequencies, in accordance with what is shown in Figure 5, where all frequencies were played at the same time.

Figure 9 shows the intensity of all frequencies detected in our setup for 10, 100, and 300 μW of average power. The response of the OFSI is not uniform. Namely, some frequency regions have a better response than others. It is important to note that the response around ∼11.25 kHz is significantly higher than the response at lower frequencies around 3 kHz. In addition, the lowest response occurs in the region that ranges from 16 kHz to 22 kHz, agreeing with Figure 7.

Analyzing the frequency response by region, we found that the OFSI detects all frequencies up to ∼2 kHz. However, after ∼2.5 kHz, the response of the system becomes strongly modulated, as shown in Figure 10a. In Figure 9, it appears that the interferometer has a larger response at ∼11.5 kHz. However, a zoom in the region reveals that the signal is modulated with a period of ∼50 Hz, as shown by the inset in Figure 10b, suggesting that an interference phenomenon is present. Figure 10c displays the modulated behavior at high vibration frequencies and depicts a reduction of the detected signal intensity as the frequency is increased to 21.5 kHz.

**Figure 11:**
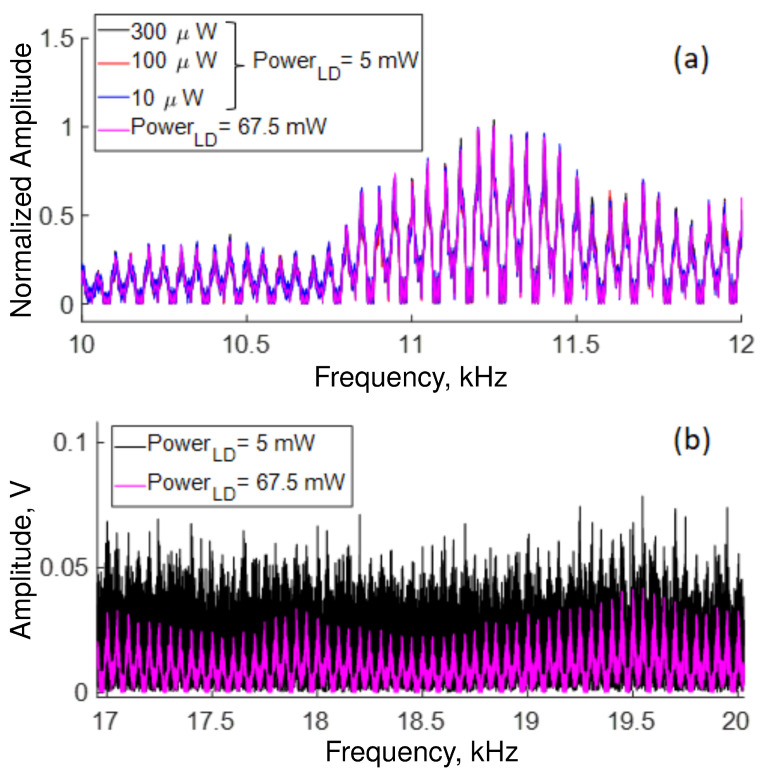
Influence on transmission response of (**a**) polarizer transmission and LD output power in the 12 kHz region, and of (**b**) LD output power at high frequencies.

Figure 11 shows the influence of power transmission through the polarizer and pump power (see Figure 1) on OFSI response at high frequencies. Figure 11a display in blue, red, and black traces the transmission normalized for 300, 100, and 10 microwatts of average output power, respectively, adjusted by QWR, and the LD output power set at 5 mW. The magenta trace was measured with the QWR adjusted for maximum transmission by the OFSI, with the LD power set at 67.5 mW. The traces of Figure 11a are almost indistinguishable, revealing that our system is highly reproducible and robust, and the period of modulated signal does not depend on the state of polarization at the input of the Sagnac interferometer. Moreover, we note that increasing the LD output power allows us to eliminate noisy signals, as shown in the traces in Figure 11b, where the signals are very noisy when the LD power is adjusted at 5 mW (black trace) in contrast with curves when the LD output power is adjusted at 67.5 mW (magenta trace) where the modulation is clearly defined.

**Figure 12:**
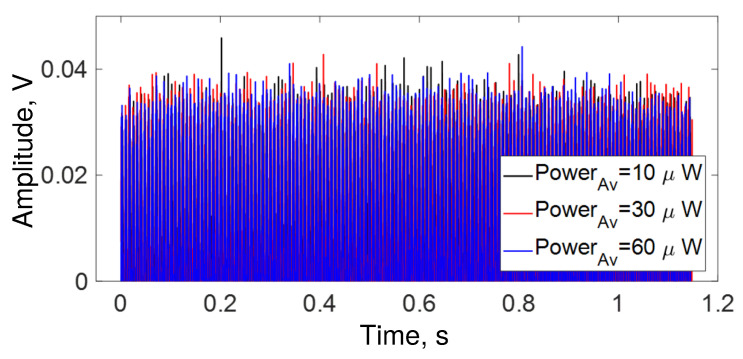
Temporal response of a 10 m loop OFSI. Blue trace = 10 μW, red trace = 30 μW, and black trace = 60 μW of average power at the output.

For comparison, Figure 12 shows the response of a 10 m OFSI (the loop consists of two 5-m pieces of fiber, right- and left-twisted at 7 turns/m) when frequencies from 10 to 22 kHz are played mixed for ∼1.2 s. It is important to mention that the maximum average power, in this case, is ∼65 μW. In contrast to Figure 4, different values of the average power in Figure 12 yield similar intensities.

**Figure 13:**
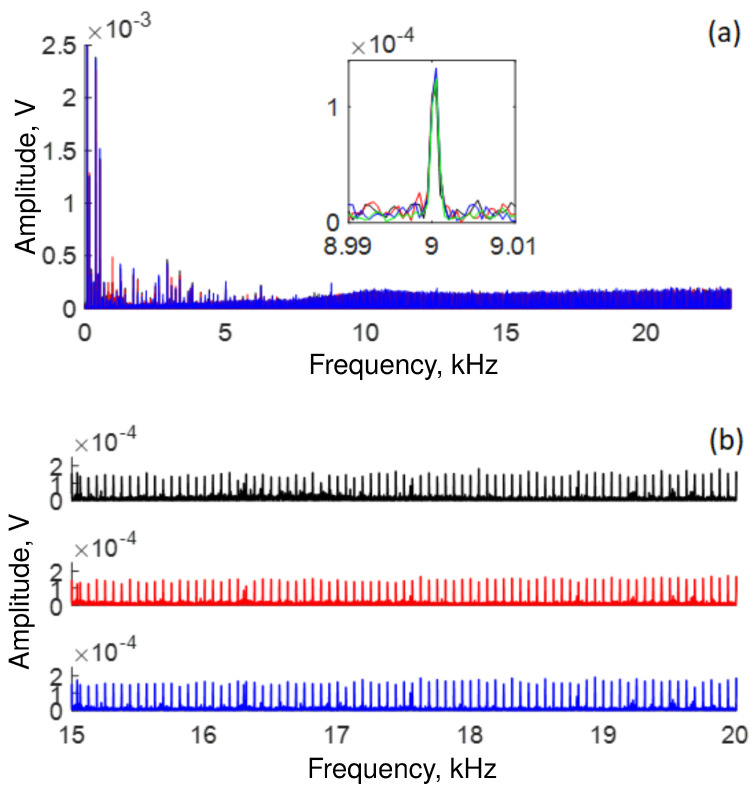
Response of a 10 m loop OFSI: (**a**) All frequencies detected during ∼2.5 s for several average output power values adjusted by the QWR (See Figure 1), inset shows a close-up on 9 kHz. (**b**) close-up on region from 15 kHz to 20 kHz. Black trace = 10 μW, red trace = 30 μW, blue trace = 60 μW.

Figure 13 shows the frequency response of the 10 m loop OFSI. Figure 13a reveals that the response at low frequencies (1.5 kHz to 5 kHz) is minor compared with Figure 5 (300 m loop OFSI) and is more uniform, presenting fewer amplitude variations with frequency. However, the response at high frequencies is low but still detectable, as shown in Figure 13b where a zoom on a range from 15 kHz to 20 kHz has been realized for the different average power values. Finally, we can appreciate that the response of the 300 m loop OFSI is better than the 10 m loop OFSI. We attribute the best results of the 300 m loop to the fact that the speaker impinges on one side of the drum, where many turns of fiber pass through, reinforcing the signal within the loop, in particular at certain frequencies where constructive interference takes place. It is relevant to mention that in our setup, with the 300 m loop OFSI, the minimum sound level detected was 56 dB.

## 4. Conclusions

In this study, a double-sense twisted low-birefringence fiber is used as the sound/ vibration sensing element in a Sagnac loop structure. We study the relation between the wave retarder adjustments and the response of Sagnac for vibration frequencies ranging from 0 kHz to 22 kHz. For a 300 m loop Sagnac, two sets of experiments were carried out, playing at the same time all the sound frequencies mixed for ∼1 s (simulating an environment with various elements that generate vibrations) and a sweep of frequencies (to analyze the influence of each frequency on the Sagnac interferometer response). For both cases, frequencies mixed and sweeps, the amplitude of the transmission response is larger for a minimal average output power, in contrast, the amplitude of transmission is reduced for a maximal average power measured at the output of the system. For mixed frequencies, the results show that the response is greater for low frequencies (in the 0 kHz to ∼5 kHz range) than for high frequencies (from ∼5 kHz to ∼22 kHz). Furthermore, the results indicate that the response vanishes at some particular frequencies. For a sweep of frequencies, the results reveal that the interferometer is sensitive to all frequencies up to ∼2 kHz. However, beyond ∼2.5 kHz, the system clearly displays a modulated response with a period of ∼50 Hz. The results on the influence of the power transmission through the polarizer and pump power on OFSI response at high frequencies reveal that our system is highly reproducible and robust, and modulation does not depend on the state of polarization at the input of the Sagnac interferometer. Additionally, increasing the pump power from 5 mW to 67.5 mw allows us to eliminate noisy signals. On the other hand, by comparing the performances of the 300 m OFSI with the experimental results of a 10 m loop OFSI, we observe that response of the latter at low frequencies (1.5 kHz to 5 kHz) is minor compared to the 300 m loop OFSI. This can be understood by considering that the speaker impinges on one side of the drum, where several turns of fiber pass through, reinforcing the signal at a larger number of points along the fiber in the case of a longer loop. Although the response at high frequencies is low, it is still detectable, making it possible to adjust the response of the vibration sensor by varying the Sagnac loop length. In summary, we have shown that it is possible to develop an OFSI with double-sense twisted low-birefringence standard fiber that can be used as highly reproducible, robust, and independent of the state of polarization to measure sound/vibrations. Moreover, the robustness, high stability, and reproducibility of the setup allow its application in numerous scientific or industrial environments.

Finally, a few technical issues, some of them related to the non-ideal conditions of the present experiments, should be briefly mentioned here, as they could affect the OFSI response and be responsible for some of its features reported here. First, since only one speaker was used to produce all frequencies in the 20 Hz to 20 kHz range, one can expect that its frequency response is not perfectly flat over the whole spectrum, which in turn would alter the frequency response of the OFSI. A second issue is related to the possibility of aliasing and noise effects at the detection end. The photodetector has a large bandwidth (5 GHz), and no anti-aliasing filter at half the sampling rate (100 kS/s) was used, meaning that all noise above that frequency would appear on the FFT at lower frequencies. Hence it cannot be excluded that some of the spectral features reported here are actually aliases of higher-frequency noise. On the other hand, some features, such as the modulation at 50 Hz, could be related to electric interference. All these elements should be taken into account in the future, a more detailed study of the OFSI to be used as a vibration sensor. In addition, a careful analysis of the acoustic coupling from air to the fiber should be carried out (which depends not only on fiber length and the number of turns, as already mentioned, but also on several other factors, such as the drum characteristics and details of fiber wrapping), to tailor a frequency response as flat as possible.

## Data Availability

Data underlying the results presented in this paper are not publicly available at this time but may be obtained from the authors upon reasonable request.

## Abbreviations

The following abbreviations are used in this manuscript:

DAQ	Data Acquisition
DOFS	Distributed Optical Fiber Sensor
FRL	Fiber Ring Laser
HWR	Half Wave Retarder
LD	Laser Diode
OFSI	Optical Fiber Sagnac Interferometer
OTDR	Optical Time Domain Reflectometry
PC	Polarization Controller
PM	Polarization Maintaining
QWR	Quarter Wave Retarder
